# Shear wave elastography for assessing kidneys in pediatric patients with primary nephrotic syndrome

**DOI:** 10.1186/s13244-025-02070-x

**Published:** 2025-08-23

**Authors:** Jianhuan Yang, Fangfang Yu, Dexuan Wang, Maosheng Xu, Hongxia Luo

**Affiliations:** 1 Department of Pediatric Nephrology, The Second Affiliated Hospital and Yuying Children’s Hospital of Wenzhou Medical University, Wenzhou, China; 2 Department of Ultrasonic Diagnosis, The Second Affiliated Hospital and Yuying Children’s Hospital of Wenzhou Medical University, Wenzhou, China; 3 Department of Key Laboratory of Structural and Functional Imaging of Wenzhou, Wenzhou, China

**Keywords:** Shear wave elastography, Ultrasound, Primary nephrotic syndrome, Pediatric, Renal fibrosis

## Abstract

**Objective:**

Primary nephrotic syndrome (PNS) is a prevalent kidney disorder in pediatric patients, characterized by significant proteinuria, hypoalbuminemia, and edema, which poses serious health risks and economic burdens due to frequent relapses and hospitalizations. This study aims to explore the utility of shear wave elastography (SWE) as a novel, non-invasive biomarker for assessing renal health in this population.

**Materials and methods:**

We used a cross-sectional design involving 76 pediatric patients with PNS and a control group, and measured renal stiffness through SWE The clinical characteristics of the nephrotic group were collected, including age, sex, disease duration, clinical type, 24-h urine protein, plasma albumin, and the relationship with 2D-SWE value was analyzed.

**Results:**

Our results demonstrated a significant difference in renal elasticity, with the nephrotic syndrome group exhibiting a mean shear wave velocity (YM) of 22.36 ± 8.53 kPa compared to 17.51 ± 4.09 kPa in controls (*p* < 0.05). Furthermore, the area under the ROC curve for SWE was 0.67, indicating moderate predictive capability for renal damage. Notably, there were no significant differences in YM values across various clinical classifications of nephrotic syndrome, suggesting a uniform renal damage assessment irrespective of clinical type. Additionally, renal elasticity did not significantly vary regardless of whether the patient’s proteinuria had improved. (*p* = 0.464), indicating SWE’s potential as an independent biomarker.

**Conclusions:**

Our findings highlight the promise of SWE in enhancing diagnostic accuracy and prognostic evaluation in pediatric nephrotic syndrome.

**Critical relevance statement:**

Shear wave elastography is a valuable noninvasive method for assessing renal elasticity in children with primary nephrotic syndrome.

**Key Points:**

- Shear wave elastography (SWE) can be used to evaluate the elasticity of renal tissue.
- SWE values were higher in children with PNS than in the control group.
- SWE is a valuable non-invasive method for assessing renal elasticity in children with PNS.

**Graphical Abstract:**

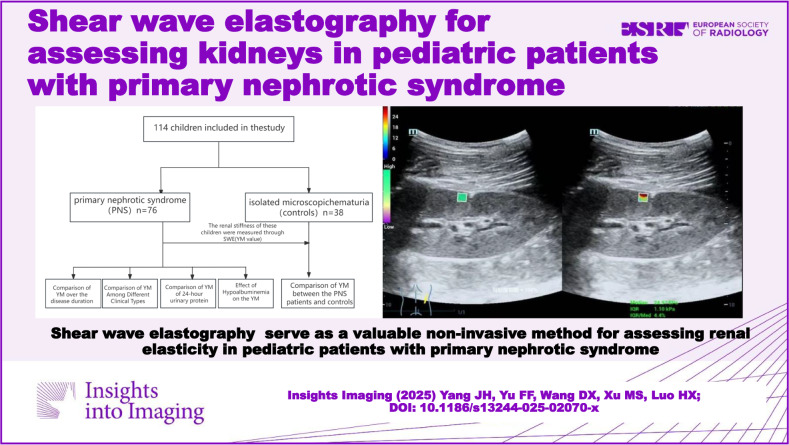

## Introduction

Primary nephrotic syndrome (PNS) is the most common type of nephrotic syndrome in children, and its incidence was estimated at 2–7/100,000 children, with the prevalence 16/100,000 [1]. Most patients with PNS are sensitive to steroids, but 10% of patients are resistant to steroids, with 50% of them progressing to end-stage renal disease (ESRD) during long-term follow-up [2]. Focal segmental glomerulosclerosis (FSGS) is the most common pathological type of steroid resistance, which can cause glomerulosclerosis, and renal biopsy is the gold standard for its diagnosis [3, 4]. However, renal puncture is an invasive procedure that cannot be widely used in the early diagnosis of nephrotic syndrome. Therefore, a safe, non-invasive and simple method is needed to evaluate the changes of renal texture in patients with nephrotic syndrome at an early stage.

Ultrasound shear wave elastography (SWE) is a non-invasive technique developed in recent years to detect tissue elasticity, which can be evaluated by measuring Young’s modulus (YM). SWE has been widely studied in the diagnosis of liver fibrosis, thyroid and breast diseases in recent years [5–7]. It was reported that SWE can be used to evaluate the elasticity of renal tissue and can be used as a non-invasive examination method to predict renal fibrosis [8, 9]. Few studies have been reported on the renal elasticity in children with nephrotic syndrome.

The primary objective of this research is to establish the clinical significance of renal elasticity as measured by SWE in pediatric patients with PNS. This study adopted a cross-sectional design to assess renal stiffness in pediatric patients diagnosed with PNS using SWE. By comparing the renal stiffness measurements of patients with PNS to those of a control group, the study aims to elucidate the relationship between renal elasticity and various clinical parameters, including age, sex, disease duration, and proteinuria levels. The integration of innovative imaging techniques into clinical practice could facilitate earlier diagnosis and more effective management of PNS, ultimately improving quality of life and health outcomes for affected children [10, 11].

## Materials and methods

This study was conducted in accordance with the Declaration of Helsinki and approved by the medical ethics committee of the Second Affiliated Hospital of Wenzhou Medical University (approval number: 2022-K-314-02). Written informed consent was obtained from all participants.

### Study design and participants

This study adopts a cross-sectional design. Data were obtained from 76 patients with PNS diagnosed in the Department of Pediatric Nephrology of the Second Affiliated Hospital of Wenzhou Medical University from January 2022 to January 2024.

The diagnosis of nephrotic syndrome was based on proteinuria > 50 mg/kg/24 h, hypoalbuminemia (< 25 g/L), hyperlipidemia, and edema. Relapse of PNS was defined as a recurrence of nephrotic range proteinuria, and remission as 0 to trace proteinuria in urinalysis or 24-h urinary collection ≤ 0.15 g/day for 3 consecutive days. Based on the response to steroids, the disease was categorized as steroid-sensitive nephrotic syndrome (SSNS), defined as remission of nephrotic syndrome within 4 weeks of steroid therapy; steroid-dependent nephrotic syndrome (SDNS), defined as nephrotic syndrome with 2 consecutive relapses during or within 2 weeks of ceasing steroid therapy; and steroid-resistant nephrotic syndrome (SRNS), defined as no remission of nephrotic syndrome despite 4 weeks of steroid therapy; frequently-relapsing nephrotic syndrome (FRNS) as nephrotic syndrome with two or more relapses within 6 months of the initial response, or with four or more relapses during any 12-month period [12]. The control group comprised 38 subjects diagnosed as isolated microscopic hematuria in our department in the same period. Isolated microscopic hematuria is defined as more than 3 RBC/high-power field by urine microscopy examination of freshly voided urine, without any clinical symptoms or signs.

2D-SWE values were detected in all participants, including nephrotic group and control group. The clinical characteristics of the nephrotic group were collected, including age, sex, disease duration, clinical type, 24-hour urine protein, plasma albumin, and the relationship with 2D-SWE value was analyzed.

### Shear wave elastographic examinations

**Fig. 1:**
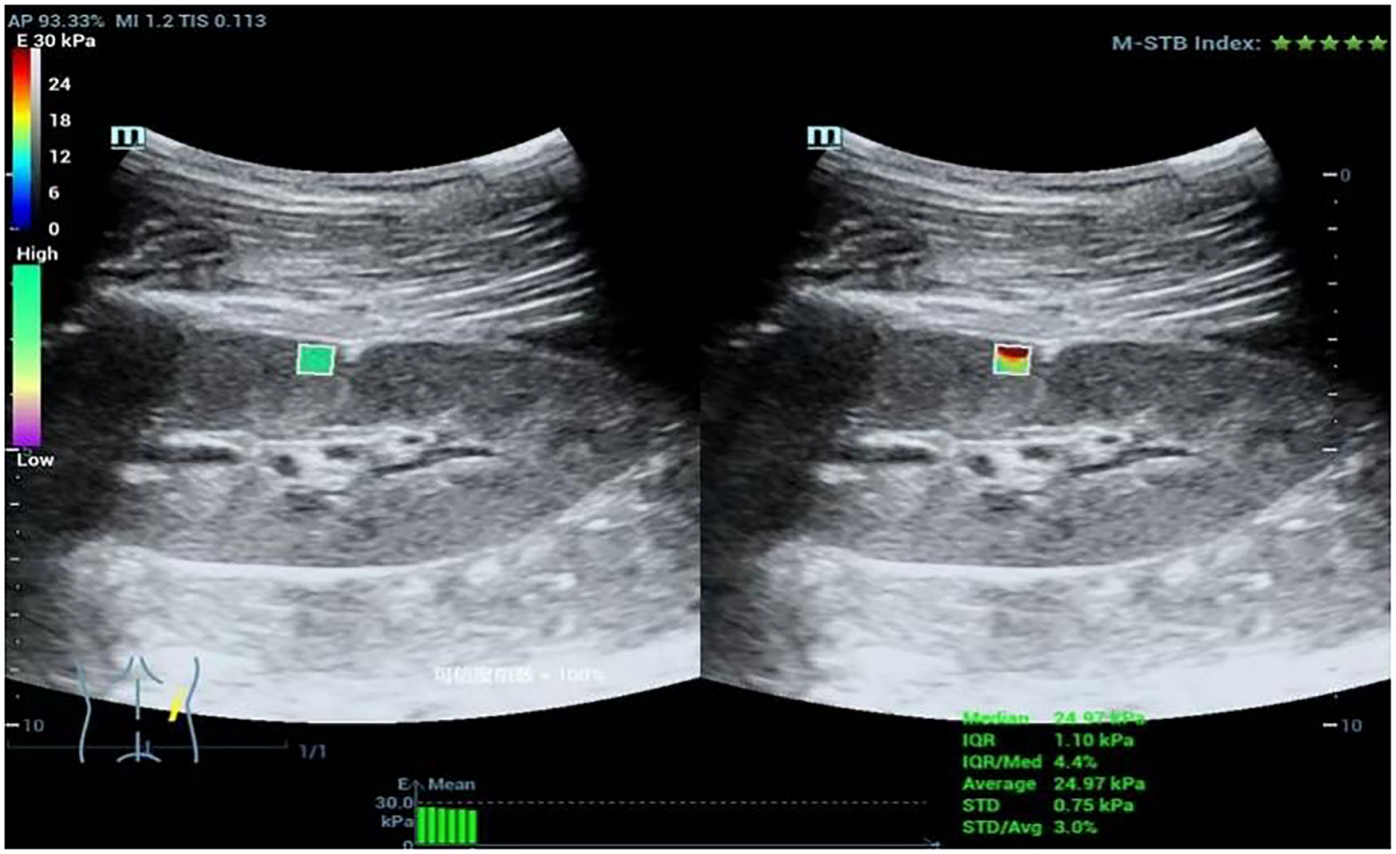
Ultrasound elastography in the PNS patients

The elasticity of renal tissue was measured by SWE in STQ mode, and the results were expressed by Young’s modulus (YM). The Mindray Resona 7-T ultrasonic device was used, equipped with an SC5-1U convex array probe. All images were obtained from the patients in the lateral decubitus position while holding their breath by two skilled radiologists (Y.F.F. and L.H.X., with 18 years of experience in US imaging). The sample box was placed in the right renal cortices of the middle portions (Fig. 1). A region-of-interest (ROI) of fixed size was chosen for each measurement. The motion stability (M-STB) index located in the upper right corner of the screen should be no less than four stars. The final elasticity values represent the mean of six measurements, with the interquartile range (IQR) to median ratio (IQR/M) between measurements less than 30% (calculated automatically by Mindray Resona).

### Statistical analysis

Statistical analysis was performed using SPSS 19.0 (IBM SPSS Inc.). Continuous data were expressed as the mean (± standard deviation). Categorical variables were reported as frequencies with percentages. Two groups of continuous data were compared by a *t*-test, and multiple groups of continuous variables were tested by one-way ANOVA. Proportions were compared between the groups using the Pearson chi-squared test. Assess the diagnostic performance of SWE for nephrotic syndrome by receiver operating characteristic (ROC) curves. *p* < 0.05 was accepted as statistically significant.

## Results

76 patients with PNS and 38 age- and sex-matched controls were enrolled in the study. There was no significant difference between the two groups in terms of the mean age (8.53 ± 4.13 vs 7.58 ± 3.14 years, *p* = 0.216) and sex ratio (M: F 56/20 vs. 27/11, *p* = 0.766).

Among 76 patients with PNS, 36 cases had a course of disease less than 1 year, 19 cases between 2 and 5 years, and 21 cases more than 6 years. Among them, there were 19 first-time onset patients, 25 cases of recurrent nephrosis, and 32 cases were refractory nephrosis, including 29 cases of FRNS or SDNS, and 3 cases of SRNS. Despite their varying disease durations, all these patients had normal renal function. (Table 1).

**Table 1:** Demographic and clinical characteristics of the study and control group

	PNS patients (*n* = 76)	Controls (*n* = 38)
Ages (years)	8.53 ± 4.13	7.58 ± 3.14
Sex (%)		
Male	56 (73.68%)	27 (71.05%)
Female	20 (26.32%)	11 (28.95%)
Disease duration (years)		
≤ 1	36	--
2–5	19	
> 5	21	
Clinical type		
Incipient nephropathy	19	--
Relapse	25	
FRNS/SDNS/SRNS	32	

### Comparison of YM between the PNS patients and controls

**Fig. 2:**
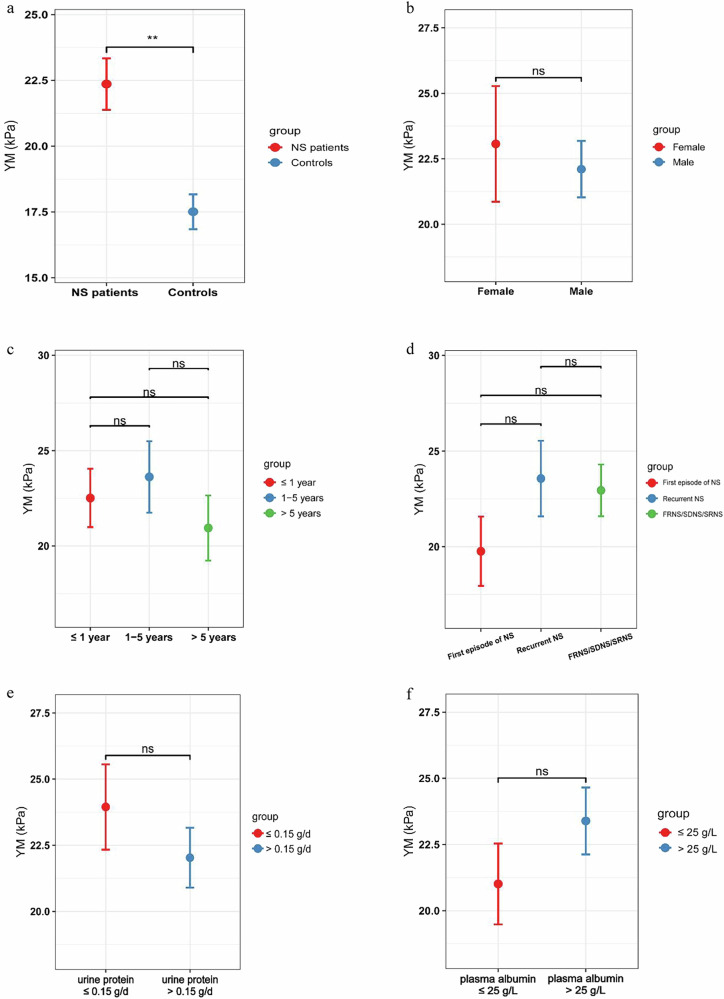
**a** Comparison of YM between the PNS patients and controls. **b** Figure 2b. Effect of sex on the YM. **c** Comparison of YM over the disease duration. **d** Comparison of YM Among Different Clinical Types of NS. **e** Comparison of YM of 24-hour urinary protein. **f** Effect of Hypoalbuminemia on the YM

The YM value in the PNS group was 22.36 ± 8.53 kPa, compared to 17.51 ± 4.09 kPa for the control group. There was a statistically significant difference between the two groups (*p* = 0.001), indicating that renal tissue elasticity is higher in patients with nephrotic syndrome than in the control group (Fig. 2a).

### Effect of sex on the YM

The YM value of 56 male patients was 22.10 ± 8.07 kPa, while the YM value was 23.07 ± 9.89 kPa in 20 female patients. There was no significant difference between the two groups (*p* = 0.067) (Fig. 2b).

### Comparison of YM over the disease duration

The YM value was 22.52 ± 9.18 kPa in 36 patients whose course of disease was less than 1 year. The course of disease was 2–5 years in 19 cases, and the YM value was 23.62 ± 8.16 kPa. The YM value of 21 patients with disease duration longer than 6 years was 20.94 ± 7.83 kPa. There was no significant difference among these three groups (*p* = 0.610). Therefore, the duration of nephrotic syndrome may be unrelated to YM values (Fig. 2c).

### Comparison of YM among different clinical types of NS

There were 19 patients with a first episode of NS, whose YM value was 19.77 ± 7.91 kPa. The YM value was 23.57 ± 9.87 kPa in 25 patients with recurrent NS. The YM value of 32 patients with refractory nephrosis (FRNS/SDNS/SRNS) was 22.95 ± 7.65 kPa. There was no significant difference among the three groups (*p* = 0.303). The results suggest that there is no difference in YM values among patients with nephrotic syndrome, whether they are experiencing a first episode or a recurrence, or whether they are steroid-sensitive or resistant (Fig. 2d).

### Comparison of the YM of 24-hour urinary protein

Among the 76 PNS patients, some experienced symptom relief due to the use of corticosteroids or a combination with immunosuppressants. 13 patients had negative 24-hour urinary protein (≤ 0.15 g/day) upon admission, while 63 patients exhibited varying degrees of proteinuria (> 0.15 g/day). We analyzed the impact of proteinuria on the YM value. The results showed that the YM value for the proteinuria-negative group was 23.95 ± 5.81 kPa, and for the proteinuria-positive group, it was 22.09 ± 8.99 kPa. There was no significant difference between the two groups (*p* = 0.464). Therefore, renal elasticity did not significantly vary regardless of whether the patient’s proteinuria had improved. (Fig. 2e).

### Effect of hypoalbuminemia on the YM

76 patients with PNS showed hypoalbuminemia of different degrees, including 33 patients with plasma albumin ≤ 25 g/L and 43 patients with plasma albumin > 25 g/L. The YM values were 23.39 ± 8.28 kPa in the former group and 21.01 ± 8.77 kPa in the latter group. There was no significant difference between the two groups (*p* = 0.23). Therefore, the degree of hypoalbuminemia is not related to the YM value (Fig. 2f).

### ROC curve of YM

**Fig. 3:**
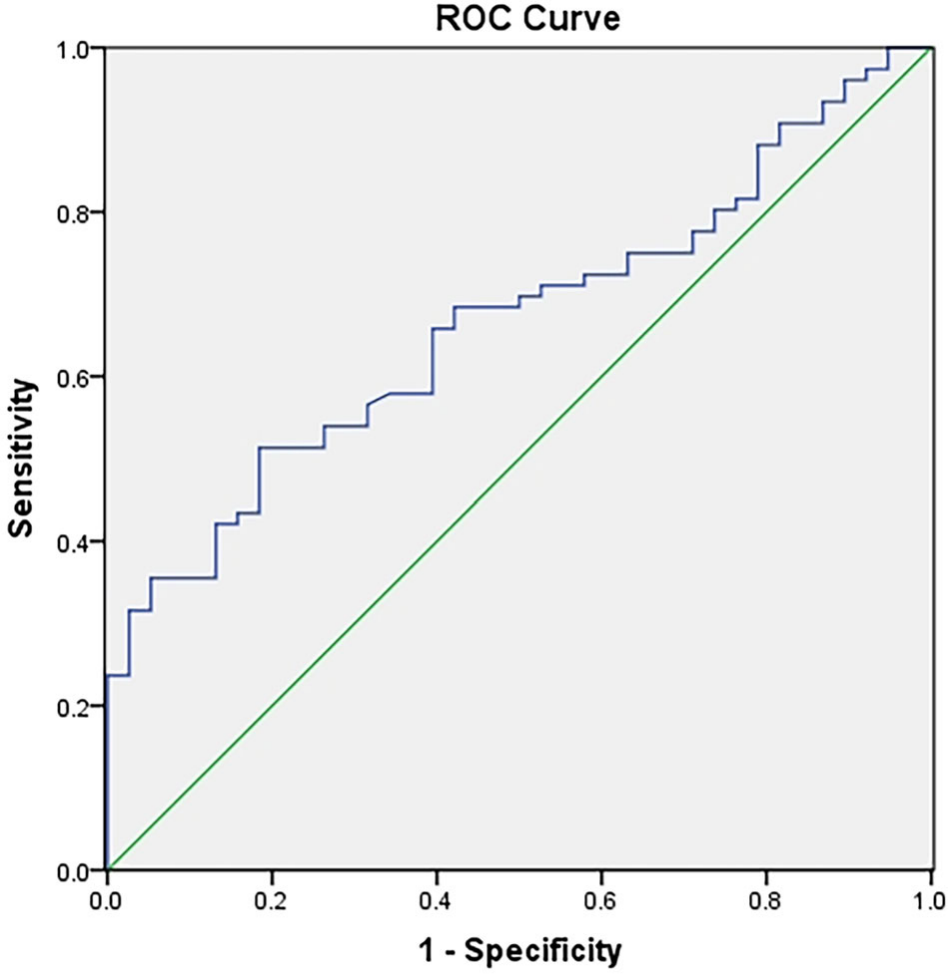
ROC curve of YM

The area under the ROC curve for SWE is 0.67. The cut-off value is 18.86 kPa, which can be used to predict renal damage in patients with nephrotic syndrome. The sensitivity is 63.2%, and the specificity is 60.5%. (Fig. 3).

## Discussion

Primary nephrotic syndrome is a significant pediatric kidney disorder characterized by substantial proteinuria, hypoalbuminemia, and edema, leading to severe health risks and long-term complications in affected children [13, 14]. This condition not only impacts the physical well-being of pediatric patients but also imposes considerable economic burdens on healthcare systems due to recurrent relapses and hospitalizations. Current diagnostic methods, such as urinalysis, and treatments like steroid therapy, exhibit limitations, including delayed diagnosis and variable treatment responses, underscoring the necessity for innovative assessment approaches to improve patient management.

This study investigates renal elasticity through SWE as a potential novel biomarker for evaluating kidney health in pediatric patients with PNS. By employing a cross-sectional design, we aim to compare renal stiffness measurements between patients and a control group. The findings from this research are anticipated to provide insights into the associations between renal elasticity and various clinical characteristics.

In this study, we detected the renal elasticity in children with PNS by use of ultrasound SWE. Compared to patients with isolated hematuria, patients with nephrotic syndrome exhibited higher YM values, which may be related to the long-term persistent proteinuria. The presence of albuminuria or proteinuria constitutes a sign of kidney damage and, together with the estimation of glomerular filtration rate, is based on the evaluation of chronic kidney disease [15]. Mesangial toxicity from specific filtered compounds such as albumin-bound fatty acids and transferrin/iron, tubular overload and hyperplasia, and induction of proinflammatory molecules such as MCP-1 and inflammatory cytokines are some of the proposed mechanisms [16, 17]. Therefore, persistent proteinuria may lead to kidney damage and fibrosis, causing changes in renal tissue elasticity and variations in YM values.

Shear wave elastography accurately detects chronic renal damage resulting from glomerular sclerosis, interstitial fibrosis and tubular atrophy [18]. Patients with no glomerular sclerosis showed lower mean YM measurements compared to those with glomerular sclerosis [19, 20]. However, some studies found no correlation between elasticity and renal fibrosis [21]. A meta-analysis of nine study parts from six studies was performed [22]. The studies proved very heterogeneous in terms of design and results. The shear wave velocity difference of −0.82 m/s between CKD patients and controls was not significant.

Our team previously found that [23] changes in renal elasticity contribute to the detection of early renal injury in patients with hematuria and/or proteinuria. The renal cortical stiffness in patients with proteinuria presenting with hematuria is abnormally reduced. In this study, we found that SWE can be used to predict early renal damage in patients with nephrotic syndrome, with a cut-off value of 18.86 kPa, a sensitivity of 63.2%, and a specificity of 60.5%. Due to the long-term and recurrent proteinuria, which can easily lead to renal tissue damage, patients with nephrotic syndrome have higher YM measurements than those with isolated hematuria, indicating reduced kidney elasticity. However, the area under the curve value in this study was 0.67, suggesting a moderate ability to predict renal impairment. Because of its non-invasive nature, SWE can monitor changes in renal tissue elasticity, serving as a supplementary method for early prediction of renal damage. However, it should be noted that it cannot replace the gold-standard method for renal pathology diagnosis.

YM values are influenced by various factors, such as age, gender, weight, etc. However, this study found that the YM value is not related to the gender, duration of disease, clinical type, 24-h proteinuria and plasma albumin in patients with nephrotic syndrome. We found that although the enrolled patients with nephrotic syndrome had varying disease durations, varying responses to steroids and frequencies of relapse, statistical results indicated that the YM value was not correlated with the duration of the disease or clinical type. This may be because the majority of pediatric nephrotic syndrome patients have a good prognosis. Unlike adult patients with nephrotic syndrome, the majority of pediatric cases involve minimal change disease and are responsive to steroid treatment [24]. Although about half of the children may experience relapses during steroid therapy, most patients have a good prognosis and rarely progress to chronic kidney failure [25, 26].

We found that the YM values in patients with nephrotic syndrome were not correlated with urinary protein and plasma albumin levels. Although nephrotic syndrome is characterized by significant proteinuria and hypoalbuminemia, the prognosis for pediatric nephrotic syndrome is notably better than that of adult nephrotic syndrome due to the fact that 80-90% of children with nephrotic syndrome respond well to steroid therapy and can achieve rapid remission [27]. Thus, short-term proteinuria and hypoproteinemia have a minimal impact on renal damage.

This study has several limitations that warrant consideration. Firstly, the relatively small sample size may limit the statistical power and generalizability of our findings. Additionally, the cross-sectional design restricts the ability to establish causal relationships between renal elasticity and clinical outcomes over time. Since the inpatient department could not obtain data from a normal group, we selected patients with hematuria as the control group, which may have subclinical pathologies affecting elasticity. The lack of long-term follow-up data means we cannot assess the prognostic significance of SWE in predicting disease progression or renal function decline in pediatric patients. Furthermore, while SWE shows promise as a non-invasive biomarker, the absence of experimental validation raises questions about its applicability in routine clinical practice. These limitations highlight the necessity for future research to incorporate larger cohorts and longitudinal data to substantiate the clinical utility of this innovative diagnostic tool.

## Conclusion

Our study provides compelling evidence that SWE may serve as a valuable non-invasive method for assessing renal elasticity in pediatric patients with PNS. The significant differences observed in renal stiffness between nephrotic syndrome patients and controls suggest that SWE could enhance diagnostic accuracy and inform treatment strategies.

## Data Availability

The data in this study involve personal privacy, and anonymized data can be requested from the corresponding author if there are reasonable reasons.

## Abbreviations

FRNS: Frequently-relapsing nephrotic syndrome
PNS: Primary nephrotic syndrome
ROC: Receiver operating characteristic
SDNS: Steroid-dependent nephrotic syndrome
SRNS: Steroid-resistant nephrotic syndrome
SWE: Shear wave elastography
YM: Young’s modulus
